# A New Remedy for Hydrophobia

**Published:** 1880-08

**Authors:** 


### EXCERPTA.

A New Remedy for Hydrophobia.—The author makes known a plant which enjoys a great reputation for the cure of hydrophobia in the kingdom of Annam. This plant, called hoang-nan, is a species of convolvulus, which is closely related to false angustura; its effects should be analogous to those of strychnia and brucia. M. Bouley, in speaking of this new remedy in the *Recueil de Med. Veterinaire*, regrets that no case has been cited demonstrative of its efficacy; but thinks that experiments should be made. M. Bouley relates an anecdote apropos of garlic, a substance which enjoys a great reputation among anti-hydrophobic remedies, and which is an ingredient of a good many formulæ long kept secret. A young man had been bitten by a mad dog and symptoms of hydrophobia soon manifested themselves. The affrighted family, not knowing what to do with the unfortunate, shut him up in a small chamber in which garlic had been placed to dry. In his fury the unfortunate man threw himself upon the bundles of garlic, ate of them abundantly, and soon became drowsy and fell into a profound sleep. On awakening he was cured; the symptoms of hydrophobia had entirely disappeared.—*M. Lesserteur in Journ. de Med., May*, 1880.

